# Supplementary material for: Prevalence, causes and impact of musculoskeletal impairment in Malawi: A national cluster randomized survey
Source: PLoS One. 2021 Jan 6;16(1):e0243536. doi: 10.1371/journal.pone.0243536 (PMC7787380; doi:10.1371/journal.pone.0243536)
Supplement: S1 File — (PDF) [file pone.0243536.s001.pdf]

☐ If this box is marked then complete a full assessment on this individual.

RAPID ASSESSMENT OF MUSCULOSKELETAL IMPAIRMENT

A. GENERAL INFORMATION

Year - month: ☐☐☐☐ - ☐☐

Cluster: ☐☐☐ Household Number: ☐☐ Individual no: ☐☐ Age (years): ☐☐

Examination status:
- Examined: O (1)
- Not available: O (2) — Go to Section G
- Refused: O (3) — Go to Section G
- Unable to communicate: O (4) — Go to Section G

Sex: Male: O (1) Female: O (2)

Location: Rural: O (1) Urban: O (2)

Level of education of head of household:
- None: O (1)
- Primary: O (2)
- Secondary: O (3)
- University: O (4)

B. SCREEN FOR MUSCULOSKELETAL IMPAIRMENT

Screen by eligible person: O (1)
Screen by proxy: O (0)

Use this prefix for 5 and under (by proxy): Compared to other children

	Yes	No
1. Is any part of your body missing or misshapen?:	O	O
2. Do you have any difficulty using your arms?:	O	O
3. Do you have any difficulty using your legs?:	O	O
4. Do you have any difficulty using any other part of your body?:	O	O
5. Do you need a mobility aid or prosthesis?:	O	O
6. Do you have convulsions, involuntary movement, rigidity or loss of consciousness?:	O	O

**Duration**	yes	no
Has it lasted>1m?	O	O
Is it permanent?	O	O

Screen Case: O (1)
Not Screen case: O (0)

C. OBSERVATION OF ACTIVITIES

		Yes	No
Position	Squat/sit bending knees:	O	O
	Stand up straight on natural legs:	O	O
	Hold arms straight above head, fingers straight:	O	O
Mobility	Walk along the 11 metre rope:	O	O
	Do it in less than 10 secs:	O	O
	Do it without limping:	O	O
Right hand function	Touch Nose:	O	O
	Pick up coin and put in cup:	O	O
	Tip coin into bowl:	O	O
Left hand function	Touch Nose:	O	O
	Pick up coin and put in cup:	O	O
	Tip coin into bowl:	O	O

D. SEIZURE HISTORY

No history of seizure: O (0)
History of seizure: O (1)

3 or more seizures

Number of episodes in last year:
- 0: O (1)
- 1-2: O (2)
- 3-10: O (3)
- >10: O (4)
- Not applicable (never had seizure): O (5)

Type of seizure (tick one only)
- Absences: O (1)
- Convulsions: O (2)
- Not applicable (never had seizure): O (3)

E. DURATION AND CONSANGUINITY

Age at impairment:
- Since birth: O (1)
- after birth-1 year: O (2)
- 1-5 years O (3)
- 6-15 years: O (4)
- 16-39 years: O (5)
- >40 years: O (6)
- Not applicable (No impairment:) O (7)

	yes	no
Consanguinity:	O	O

F. AETIOLOGY

Tick one only for each impairment

	Impairment no: 1	2	
Family history:	O	O	(1)
Congenital but no family history:	O	O	(2)
Perinatal hypoxia:	O	O	(3)
Trauma — War:	O	O	(4)
Trauma — 94 war:	O	O	(5)
Trauma — RTA:	O	O	(6)
Trauma — Civil violence:	O	O	(7)
Trauma — Domestic violence:	O	O	(8)
Trauma — Deliberate self harm:	O	O	(9)
Trauma — Other inc accidents:	O	O	(10)
Developmental / Nutritional:	O	O	(11)
Infection:	O	O	(12)
Neoplasm:	O	O	(13)
Iatrogenic:	O	O	(14)
Traditional	O	O	(15)
Unknown:	O	O	(16)
Other:	O	O	(17)
Not applicable (No impairment:)	O	O	(18)

Specify……………………………….

G. HISTORY IF NOT EXAMINED

Subject believed to be:
- Not MSI impaired: O (1)
- MSI impaired with treatment: O (2)
- MSI impaired without treatment: O (3)
- Not applicable (examined): O (4)
