# Supplementary material for: Prevalence, causes and impact of musculoskeletal impairment in Malawi: A national cluster randomized survey
Source: PLoS One. 2021 Jan 6;16(1):e0243536. doi: 10.1371/journal.pone.0243536 (PMC7787380; doi:10.1371/journal.pone.0243536)
Supplement: Attachment — Submitted filename: Response To Reviewers.docx [file pone.0243536.s004.docx]

Dear Editor,

Thank you for the opportunity to resubmit this article after revisions, following a brief feedback from you.

We thank you and the reviewers for your time and efforts to provide helpful comments, and have tried to address all these below and through the revisions in the manuscript:

ACADEMIC EDITOR: In abstract, the methods part, please explain briefly how the study subjects were randomized and how musculoskeletal impairments were measured in ICF criteria and the main statistical methods used to analyse the data.

The abstract has been completely revised and most of the points raised were addressed.

Thank you again for the opportunity to resubmit this manuscript and many thanks to the reviewers for their positive response to our previous manuscript. 

Best regards
Leonard Banza
